# A qualitative study of the determinants of adherence to NICE falls guideline in managing older fallers attending an emergency department

**DOI:** 10.1186/s12245-018-0192-9

**Published:** 2018-07-18

**Authors:** Helen McEwan, Richard Baker, Natalie Armstrong, Jay Banerjee

**Affiliations:** 1 Department of Psychology, Faculty of Business, Law and Social Sciences, Birmingham City University, C303 Curzon Building, 4 Cardigan Street, Birmingham, B4 7BD England; 2 Department of Health Sciences, Centre for Medicine, University of Leicester, Leicester, England; 3 Department of Emergency Medicine, University Hospitals of Leicester NHS Trust, Leicester, England

**Keywords:** Accidental falls, Emergency care systems, Emergency departments, Guidelines, Geriatrics, Qualitative research

## Abstract

**Background:**

The National Institute for Health and Care Excellence (NICE) 2004 Falls guideline was developed to improve the assessment and management of falls and prevention of future falls. However, adherence to the guideline can be poor. As emergency departments (EDs) are usually consulted by older adults (aged 65 and over) who experience a fall, they provide a setting in which assessments can be conducted or referrals made to more appropriate settings.

The objective of this study was to investigate how falls are managed in EDs, reasons why guideline recommendations are not always followed, and what happens instead.

**Methods:**

The study involved two EDs. We undertook 27 episodes of observation of healthcare professional interactions with patients aged 65 or over presenting with a fall, supported by review of the clinical records of these interactions, and subsequently, 30 interviews with healthcare professionals. The qualitative analysis used the framework approach.

**Results:**

Various barriers and enablers (i.e. determinants of practice) influenced adherence at both EDs, including the following: support from senior staff; education; cross-boundary care; definition of falls; communication; organisational factors; and staffing.

**Conclusions:**

A variety of factors influence adherence to the Falls guideline within an ED, and it may be difficult to address all of them simultaneously. Simple interventions such as education and pro-formas are unlikely to have substantial effects alone. However, taking advantage of the influence of senior staff on juniors could enhance adherence. In addition, collaborative care with other NHS services offers a potential approach for emergency practitioners to play a part in managing and preventing falls.

## Background

The definition of a fall as “an event whereby an individual comes to rest on the ground or another lower level with or without loss of consciousness” [1] was adopted by the National Institute for Health and Care Excellence (NICE) in its guideline on falls [2].

Approximately, one third of adults aged 65 and over fall each year [3]. A consequence of falls can be a fractured neck of femur, which has a monthly mortality rate approximately 10%, and a 1 year mortality rate of around 33% [4]. Increasing awareness of fall incidence and consequences, for both individuals and the UK NHS, is arguably a public health priority [5].

Falls account for over 600,000 emergency department (ED) attendances and lead to over 200,000 admissions annually in the UK NHS [6, 7]. However, some healthcare professionals are not aware of the role they can play in managing and preventing falls [7]. In particular, the identification of older adults at risk of falls (and repeat-falls) is required to prevent occurrence [7], and the ED may be able to facilitate this [8].

The NICE 2004 Falls guideline [2] was developed in order to reduce the impact of falls upon older adults and to limit NHS costs. The guideline was updated in 2013, although the recommendations on assessment after a fall not materially changed, and therefore, these recommendations have had ample time for them to be adopted into routine practice.

Falls are caused by various factors, all of which can be investigated through comprehensive patient assessments, which may reduce recurrent falls [9]. The guideline recommends a multifactorial falls risk assessment in older adults who have fallen (Table 1). The NICE Falls guideline applies to ED practice [2], and as EDs are usually the first, and often only, service consulted by older adults after a fall, they provide a setting in which assessments can be conducted or referrals made to a more appropriate setting [8, 10].

**Table 1:** Falls guideline recommendations [2] on multifactorial risk assessments in older adults

A falls risk assessment includes the following:	
1	Identification of falls history.
2	Assessment of gait and balance.
3	Assessment of osteoporosis risk.
4	Assessment of perceived functional ability and fear related to falling.
5	Assessment of visual impairment.
6	Assessment of cognitive impairment and neurological examination.
7	Assessment of urinary incontinence.
8	Assessment (or recommended assessment) of home hazards.
9	Cardiovascular examination.
10	Medication review.
11	Encouraged to participate in a falls prevention programme.

Our focus was on multifactorial assessments, and the degree to which these are delivered according to the guideline (i.e. adhered to).

Our theoretical framework was developed from studies of determinants of adherence to the NICE Falls guideline in an ED. Our approach to studying adherence drew on the work of Cabana et al. [9] and Flottorp et al. [11]. It applies the idea of determinants of adherence that forms part of the model of tailored implementation [12]; the premise being that interventions tailored to the prevailing determinants are more likely to be effective than interventions chosen without tailoring. We have also drawn on research evaluating barriers to implementation, and the effectiveness of multi-faceted interventions, in implementing fall prevention guidelines [7, 13–15].

The study aimed to investigate (1) how falls are managed in EDs, (2) the barriers and enablers (collectively, referred to as the determinants of practice) influencing adherence to the guideline in EDs, and (3) ways barriers could be addressed.

## Methods

Two sites were chosen to provide some variety of setting, individuals, and contexts. Recruitment from both hospitals allowed comparison of falls management in a busy city hospital ED (A) with that in a less busy town hospital ED (B). Further information is provided in Table 2, although limited in order to retain anonymity.

**Table 2:** Hospital sites - 2013

	Hospital A	Hospital B
Hospital catchment area	18.3 million emergency department attenders in 2013 in England.	
	Catchment area of approximately 1.1 million people.	Catchment area of between 450,000 and 650,000 people.
Hospital size	More than 1500 beds.	Less than 700 beds.
Number of ED attenders	Between 130,000 and 150,000 emergency department attenders. January–December 2013. 400–500 patients seen per day.	Between 80,000 and 100,000 emergency department attenders. November 2012–December 2013. 100–300 patients seen per day.
City/town located:		
Average age of local population	Between 30 and 40.	Between 35 and 45.
Male/female % representation of local population	Approximately 50/50 split.	Approximately 50/50 split.
Ethnicity of local population	Between 60 and 70% born in England.	Between 80 and 90% born in England.
Level of deprivation in catchment population.	Ranked in the top 20 most deprived areas in England.	Ranked below the 140 most deprived areas in England.
Emergency department structure	The emergency departments comprised 3 sub-areas*: 1) Minors—an area in which patients with less serious injuries or illnesses were treated, 2) Majors—an area for treatment of non-ambulatory patients and those with potentially serious conditions; 3) ‘Resus’—for individuals who were seriously ill or injured.	
Standard treatment process	1) Patient presents. 2) Handover from ambulance crew (if at majors), or if at minors, present to receptionist and assigned to triage. 3) Patient triaged by an ED nurse. 4) Details input onto computer/written on whiteboard. 5) Patient seen by a junior doctor/advanced nurse practitioner (ANP), and a consultant, as required. 6) Patient receives treatment from nurse/healthcare assistant as directed, including investigations, e.g. ECG/blood tests. 7) If tests have been conducted, the results are assessed by a doctor/ANP to decide on the best treatment pathways. 8) Treated and discharged home/admitted, or transferred for further treatment (e.g. applying plaster cast) and then sent home/admitted.	
Emergency facilities for older frail patients, which they could be referred to post ED discharge*	Emergency frailty unit.	N/A

*Observations took place in major and minor injury departments only. This was to focus the research on generic care within the ED, not specialist services

Observation and semi-structured interviews were conducted at both sites, allowing adherence practices to be viewed from both the perspective of an outside observer, as well as the opinions of those working within the ED. Consent to participation in the study was sought from both ED professionals and patients presenting with a fall, participants being recruited through convenience sampling. Prior to seeking consent, potential participants were given information about the study and the characteristics of the researcher (e.g. non-clinical).

Care of patients aged 65 and over with a fall was observed from presentation at the ED, until discharge or admission to another department. The observer (HM) observed 30 professionals (20 doctors, 10 nursing staff) deliver 27 episodes of care. Of the 27 patients, 19 were male and 8 female, ranging in age from 67 to 98. Observation took place in major and minor injury departments, through watching, listening to, and recording interactions, but not participating in these [16]; Table 1 was referred to, to monitor adherence.

Contemporaneous notes were made for the duration of each patient’s ED stay; recording interactions between patients and professionals, questions asked, answers given, and the tests being conducted. Any potential barriers and enablers to guideline adherence were noted. After observing care, (HM) reviewed the patient’s clinical notes, using both sources to assess Falls guideline adherence. Observations were conducted until data saturation was reached-when the new information produced little or no change in the themes detected at the familiarisation stage of framework analysis.

Semi-structured interviews were conducted after the observation phase of data collection was completed. A range of professionals were recruited, including both doctors (20) and nursing staff (10). Sampling was opportunistic and professionals who had been observed delivering care could also be interviewed (3) were both observed and interviewed. Professionals were interviewed once, and interviews were conducted until data saturation was reached, that is, when there was repetition of interview responses.

Interviews (conducted by HM) were guided by an interview schedule (Table 3) developed from the Falls guideline recommendations and studies of adherence and falls management [1, 17–19], but flexibility was allowed based upon interviewees’ responses. The interview schedule was piloted with two research clinicians. The interviews were audio-recorded and transcribed anonymously. On average, interviews lasted 20 min.

**Table 3:** Interview schedule

Job role:	
1) Tell me about your job role. 2) What is your role with regards to emergency department care?	
Context of the emergency department:	
1) How do you find working within the emergency department?—asked in order to gain a general description with regards to any time pressure, etc. 2) Is there anything that you would change with regards to how care is managed in the emergency department?—potential barriers or enablers. 3) Do you think/in what ways do you think working within the emergency department context influences care?—as opposed to an inpatient ward, for example.	
Guidelines generally:	
1) NICE guidelines are developed to promote good health and patient care. How are guidelines followed within the emergency department?	
NICE Falls guideline:	
1) What is your role with regards to the management of falls in older adults? 2) Specific to falls in older adult guideline, what are the processes that you understand should be in place in emergency department care? 3) Do you think these falls guidelines are always put in place? 4) From your experience/opinion, what facilitates putting these falls guidelines into practice in emergency department care? 5) Do you think there are any barriers to following the falls guideline with older adult patients?-if yes: - What are the barriers you have experienced/witnessed?	
Final points:	
1) Have you got any other points you wish to add to this discussion of the management of falls in older adults within the emergency department?	

The interviews included five consultants, four registrars, five junior doctors, four Advanced Nurse Practitioners, four senior nurses, two staff nurses and six healthcare assistants. They sought to develop understanding of the patterns of care that had been observed and to gain views on determinants of guideline adherence.

Observation and interview data were combined to enable development of a single set of determinants, including those identified by both approaches and only one. The process included listing events that occurred during observations, characteristics of the environments, and people’s views, experiences and behaviours (HM) and (RB) collectively developed the themes from the data. Framework analysis was used as this suited our aim of producing practice-orientated findings to solve practical problems and generate policy [20–22].

Data were drawn together to define concepts, the nature of the phenomena under investigation, and categorise behaviours, motivations, and attitudes. We drew on emergent issues, including those raised by participants and what participants reported as, or were observed to be, determinants of adherence. The analysis consisted of coding transcripts and notes and then organising these into themes. Framework analysis’ five stages were adopted:

(i) Familarising ourselves with the data; (ii) identifying a thematic framework through drawing on a priori themes; topic aims, existing models of adherence [9, 11], and the interview schedule; (iii) indexing (linking data with themes); (iv) charting (providing evidence for each theme in a table); and (v) mapping and interpretation (drawing themes together, marrying up the findings from both phases of data collection, and mapping into super-ordinate and sub-ordinate themes-determinants of practice). Associations were analysed, looking for explanations, and developing ideas and strategies. Analysis of observation notes and transcribed data was supported by QSR NVivo7 software.

To help identify determinants that would be most appropriate to target in interventions to improve adherence, each determinant of practice was assessed on two criteria: (1) its apparent size of effect on adherence and (2) whether it was likely to be amenable to change. This assessment relied on our own judgement, but drew on previous research on the development of tailored implementation models [12, 23].

## Results

### Levels of adherence

Overall adherence was 62% in hospital A and 64% in hospital B.^1^ For none of the 27 observed care episodes were all 11 multifactorial assessment guideline recommendations completed. In both EDs, completion ranged from 4 to 9 of the 11 recommendations. Adherence to each recommendation varied and is summarised in Table 4.

**Table 4:** The frequency in which guideline recommendations were adhered to

	Guideline recommendation number* Referring to recommendations summarised in Table 1										
	1	2	3	4	5	6	7	8	9	10	11
Number of times each guideline recommendation was adhered to (out of 27 episodes)	25	22	6	22	18	20	6	24	27	1	15
%	93	81	22	81	67	74	22	89	100	4	56

The frequency (number and percent) in which each of the guideline recommendations (as set out in Table 1) (columns) were adhered to

At both sites, the recommendation most frequently adhered to was the completion of a cardiovascular examination (27/27 occasions). Assessments of osteoporosis risk and urinary incontinence were both completed less than a quarter of the time. A medication review was completed least frequently (1/27 occasions).

### Determinants of practice

**Fig. 1:**
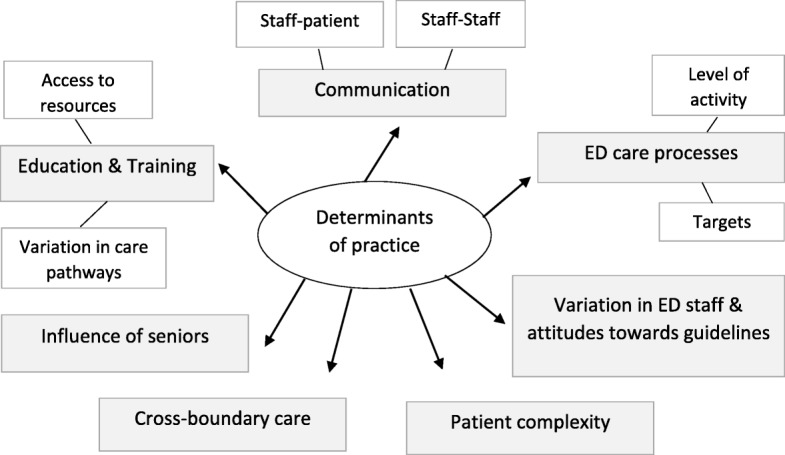
A map of the themes (barriers and enablers to adherence)

Several determinants of adherence were identified. At initial indexing, there were 17 themes, which were mapped into 7 super-ordinate and 6 sub-ordinate themes (Fig. 1).

Example quotes from observation notes and, in italics, interviews are provided, in which A/B refers to the hospital, and numbers refer to the observation/interviewee number.

#### Communication

##### Staff-patient

Professionals initially tried to talk directly to the patient, but in some circumstances, this was not possible, preventing the identification of some possible causes and consequences of falls, and ways in which to prevent re-occurrence.

##### Staff-staff

When communication within the ED and/or between departments was poor, breakdowns in care were prone to occur. Team-working could be influenced by the staff rota and the effectiveness of communication between staff.

‘Unbeknown to the professional treating the patient, they had been taken straight to an observation unit. Staff-staff member communication was a concern’.

(27- A)

“Blood tests might be omitted. You just have to hope that doctors in other area(s) will pick up on these.”

(2, Doctor, B)

#### Patient complexity

Often older adults who presented with a fall had other health problems, which sometimes took priority, making it difficult to determine causes of falls and prevent re-occurrence. There were various factors to be taken into consideration during assessment and developing treatment plans.

A patient with Alzheimer’s was found on the floor.

‘Q - (To relative) if you had the option would you rather she was kept in hospital or went home?

A - Relative stated that they needed to weigh-up the decision. With regards to Alzheimer’s they thought the patient would be better in their own environment, but with regards to their unsteadiness they would be better in hospital.’

(19 - A)

#### Education and training

Lack of education about the Falls guideline was described as influencing adherence. Some interviewees argued staff could not be familiar with all guidelines, and the department selected which to prioritise. This was due to the large number of guidelines, the large number of staff to train, and the busyness of the department.

“I don’t necessarily think some of the staff within the department understand why they are doing it. Hopefully through education you’ll get people to do it meaningfully.”

(3, Nurse, A)

Patient presented with abdominal pains after slipping.

Healthcare professional noted to (HM) that they did not think it was a fall and that it was a surgical problem; their understanding contrasted with the NICE definition.

(20 - A)

##### Variation in care pathways

Variation between professionals’ perspectives on care could affect adherence. Care pathways were viewed as a key factor.

“There are a lot of operating procedures, sometimes it varies with who’s in charge.”

(4, Doctor, A)

##### Access to resources

While there were aids to prompt falls assessments being conducted, problems in accessing the guideline were both observed and described.

“There are lots of guidance but not easily available, you have to do lots of searching.”

(6, Doctor, B)

#### Influence of seniors

Peer influence may be important. If senior members of staff were to enforce and follow guidelines, juniors may be more likely to follow suit.

“You need somebody with clinical credibility to champion work.”

(3, Doctor, A)

“My guidelines would be whatever [seniors] asked me to do.”

(7, Healthcare Assistant, B)

#### ED care processes

##### Level of activity

The busyness of the EDs and the kind of patient care provided had varied causes and implications.

“So it’s down to the ‘busyness’ of it, it’s not built for purpose, you don’t have the facilities; there’s not necessarily appropriate handovers.”

(5, Doctor, A)

‘The department was busy, consequently the professional ran out of time when conducting falls assessments and focused on the patient’s injury.’

(4 - B)

##### Targets

Professionals had competing priorities, for example, in-depth assessments, or meeting target treatment times. The 4-h target for patients to been seen and discharged from an ED [22] was a particular concern and professionals argued it could have a negative impact by not allowing time for comprehensive assessments.

“I think the four-hour target is often not conducive in providing a full assessment to somebody old.”

(3, Doctor, A)

#### Variation in ED staff and attitudes towards guidelines

Variation in staff on duty was observed to influence care, and may be associated with communication gaps. Professionals may have to adapt to working with various colleagues with different opinions or ways of working.

“If you work in a teaching hospital then guidelines are quite prominent.”

(8, Doctor, B)

The rotation of junior doctors was reported as a factor influencing care.

“Perhaps the junior doctors don’t see the value of it [guideline] because they’re not here to deal with when people get brought back in.”

(9, Nurse, A)

#### Cross-boundary care

Cross-boundary care refers to the care a patient received for their fall before or after ED discharge, and it was thought to influence care. The ED may more easily care for a patient with a fall if they have received adequate support before ED presentation and the patient is willing to accept advice. Likewise, they may more easily care for a patient if resources are in place to meet their requirements post-discharge or ward transfer.

Patient with multiple health problems, presented after falling in the bathroom.

‘The relative stated that they had had an assessment before which had not helped as they were given a Zimmer frame, which is not practical in the house.

The patient had also had a bad experience within the hospital previously, they were reluctant to accept recommended care.’

(6 - B)

“I think identifying somebody that is a risk of falls or is a ‘frequent faller’ is a key part, and you can utilise a community nurse.”

(10, Healthcare Assistant, B)

### Assessment of determinants

The determinants of practice were reviewed to identify those most likely to be suitable for addressing in a potential intervention, by assessing firstly, whether the determinant was likely to have a large impact on care (if the impact was thought to be small, little would be gained by addressing it), and secondly, whether it was possible, through intervention, to alter the effect of the determinant.

Three determinants were judged to be most important in influencing adherence and potentially most amenable to change.

- Support from seniors. Agreement amongst senior staff could lay the foundation for care throughout the ED.
- Education. Professionals need to be familiar with guidelines in order to adhere to them.
- Cross-boundary care. Adherence may be improved through considering care pathways and services to be used in conjunction with ED management.

An overview of all of the determinants and the reasons why they were considered more or less amenable to change is provided in Table 5.

**Table 5:** Determinants of practice and their amenability for change

Determinant:	Why it was perceived to be more/less amenable to change.
	More amenable to change:
Support from seniors	Agreement amongst senior medical and nursing staff on the management of falls could be reached in effectively led meetings, laying the foundation for care throughout the emergency department; this approach can be considered to be feasible.
Education	Healthcare professionals need to be familiar with the guideline in order to adhere to them; the delivery of education is potentially feasible. In order to adhere to Falls guideline, healthcare professionals need to have an awareness of what a fall is, care requirements, processes in place, and of the Falls guideline specifically.
Cross-boundary care (patient care both within and outside the boundary of the emergency department)	This determinant has the potential to be addressed through healthcare professionals and commissioners considering care pathways and alternative services to be used in conjunction with emergency department treatment of falls patients.
	Less amenable to change:
Definition of a fall	Categorisation of a fall at initial presentation influences patient care pathways and Falls guideline adherence. It is less amenable to change as in order for it to have the potential to improve, seniors need to be in agreement, and providing education needs to become possible. Therefore, these determinants need to be addressed first.
Communication and team-working, patient acceptance of staff recommendations.	Communication has the potential to be addressed, but it requires support from seniors, educational interventions, and/or support from cross-boundary services and the appropriate commissioning of services. Whether or not a patient is receptive to guideline care may have an effect upon preventative techniques being recommended or employed as a method of Falls guideline adherence.
Organisational factors within department organisation, high volume activity, access to resources, availability of medical records and targets.	Some organisational factors are less amendable to being addressed, because of practical issues, and because they are not under the control of the emergency department.
Staffing and consistency of care.	The large numbers of staff employed within the emergency departments often meant that healthcare professionals worked with a variety of staff across shifts, and this influenced team-working. Also, individuals working together may have had different attitudes about Falls guideline care. Due to the large numbers of staff, it would not be feasible to address this determinant in ensuring consistency in teams working together.

## Discussion

Various determinants were found to influence adherence practices, relating to knowledge, attitudes and behaviours; this supports previous research findings [9, 11]. Building on the influence of senior staff is a feasible approach to improve guideline adherence. If the importance of Falls guideline adherence is agreed by seniors, and a process to achieve consensus amongst them is effectively managed, juniors may receive more support and adherence may improve.

Education is often considered an appropriate intervention in implementing guidelines. It is feasible to deliver and may improve awareness of the Falls guideline [18, 19, 24]. However, its effects on adherence may be limited by other factors; in the absence of senior staff support, for example, education may have only limited effect on care by junior staff. Education may be a necessary preliminary, but it should be supplemented by other interventions [25].

Cross-boundary care has the potential to be addressed through professionals and commissioners considering care pathways and services to be used in conjunction with ED treatment of falls patients, and through ensuring collaborative working. EDs can be busy, and consistent adherence to all of the Falls guideline within the ED may not be practical. Some less time-sensitive guideline elements, such assessments of home hazards, may be more appropriately completed later on. The ED could fulfill the role of gatekeeper, in recognising the need for falls assessments and referring patients to services with more time for managing people with falls in accordance with the guideline. Service re-design with a collaborative approach to care may lead to streamlined referrals and more consistent falls assessments [17]. This approach reflects the issue of cross-boundary care, but rather than using a new care pathway to improve adherence in the ED, it could be used to appropriately direct aspects of care away from the department.

### Limitations

The study was undertaken in only two EDs, and therefore, the findings cannot be assumed to apply more widely within or outside the UK. However, most countries are likely to have some forms of screenings or guidelines in their ED and the results of our study might help inform the response to, or changes in, guidelines proposed or in use, for example, in Australia [13], Austria [14] and Singapore [15].

Convenience sampling in both observations and interviews allowed recruitment of a variety of individuals within the ED, but it was necessary to exclude a small number of patients from care homes who attended EDs unaccompanied. Excluding some individuals who could not give consent reduced variation in the patient sample. In addition, when it was busy, it was difficult to approach professionals to gain the necessary written consent to observe them attending a particular patient; this inevitably led to some data collection opportunities being missed.

Observation research has its strengths in avoiding reliance on self-report and is appropriate for use in various contexts [26]. However, observation does not collect information on the internal processes of decision-making. For example, it may have appeared that a professional did not complete an assessment of a patient’s balance when they had done so, but not recorded it, as they deemed them not to be at risk. Some gaps in decision-making were filled by reviewing clinical notes for records of tests that had been conducted and professionals’ interpretation of results.

Observation might have prompted some professionals to change their behaviour (the Hawthorne effect); however, in view of our findings of poor adherence to the guidelines, we think it unlikely that our presence encouraged healthcare professionals to adhere to the guidelines. In addition, the interview phase of research helped to shed light on what professionals knew about guidelines, and this could be compared to observed behaviour.

### Implications for practice

Through identifying determinants of practice, this study provides a basis for developing ways to address barriers to Falls guideline adherence. In contrast with other studies, we used both observation and interview research, providing two sources of evidence on the key determinants to be addressed by interventions.

## Conclusions

It appears that there is no ‘quick’ fix solution to overcoming barriers to Falls guideline adherence within the ED, but a planned approach to improving adherence has potential if it involves agreement and leadership amongst seniors, supported by staff education. Cross-boundary collaborative service designs also offer a potential approach.

## Abbreviations

ED: Emergency department
NICE: National Institute for Health and Care Excellence

## Footnotes

1 Fifteen episodes of observation were conducted at hospital A—there were 165 possible recommendations to adhere to (15 × 11 recommendations in Table 1). A total of 102 were adhered to (62%). Twelve episodes of observation were conducted at hospital B—there were 132 possible recommendations to adhere to (12 × 11 recommendations in Table 1). A total of 84 were adhered to (64%).
